# To empathize with a group or an individual? Investigating the role of cognitive cost and distress in empathy choice

**DOI:** 10.3389/fpsyg.2025.1519113

**Published:** 2025-05-07

**Authors:** Hajdi Moche, Ulrika Noryd, Sara Rydén, Daniel Västfjäll

**Affiliations:** ^1^Department of Behavioral Sciences and Learning, Linköping University, Linköping, Sweden; ^2^JEDI-Lab, Department of Behavioral Sciences and Learning, Linköping University, Linköping, Sweden; ^3^Decision Research, Eugene, OR, United States

**Keywords:** empathy, empathy choice, decision making, cognitive cost, distress, empathy selection task, emotion regulation

## Abstract

Why do we feel empathy in certain situations, but not others? Previous research has found that people often avoid empathizing when perceived as costly. This study aims to replicate and extend these findings by investigating whether the choice to empathize differs between a group or an individual. Following the empathy selection task paradigm, participants (*N* = 296) in a within-subject experiment made a series of free choices (empathy choice) selecting whether to engage in empathy or stay objective concerning a single individual and a group of individuals. They also rated perceived cognitive cost and distress concerning empathizing and staying objective. The results show that participants chose to remain objective more often than empathizing in the individual condition along with rating high levels of cognitive effort and distress. In contrast, participants in the group condition more often chose to empathize despite also rating it as more effortful and distressing. We discuss the importance of contextual factors as a main contributor to the difference in empathy choice between group and individual targets.

## Introduction

When do we empathize with other people and why? Although researchers across disciplines such as economics (Singer and Fehr, 2005), neuroscience (Decety, 2011), philosophy (Prinz, 2011), and psychology (Bloom, 2017) have attempted to understand these questions, there are still no clear answers. In this paper, we aim to add to the understanding of when and why people are willing to empathize with others by investigating the differences between empathizing with a single individual versus a group of individuals. Specifically, this paper aims to replicate and extend the findings of Cameron et al. (2019, 2022) who found that people often avoid empathizing when perceived as costly.

The paradigm of Cameron et al. (2019, 2022) focuses on empathy regulation, measured by the *empathy selection task* (EST) (Cameron et al., 2019). This framework revolves around the idea that empathy is a motivated phenomenon, suggesting that the motivation to empathize depends on the subjective expected value (i.e., people weigh the costs against the rewards; Cameron, 2018; Zaki, 2014). Thus, the idea is that people avoid empathy-eliciting situations when empathy is judged to cost time, money, or—most relevant for this line of research—cognitive or emotional effort. The EST is a behavioral measure designed to investigate the avoidance of empathy according to this framework. The EST uses free choice to assess the tendency to empathize versus staying objective. Participants are presented with two card decks—one representing to empathize and one representing to stay objective—over repeated trials and asked to choose which one they prefer before being shown a photo of a person. If choosing to empathize, participants are instructed to share in the experiences of the person and indicate their internal experiences. If choosing to stay objective, participants are instructed to remain detached and indicate the person’s external features. The willingness to empathize (or not) is measured as the proportion of empathy choice (vs. staying objective) in the EST.

In previous studies within this framework, the EST variations have mainly been about changes in the instructions or of the individual’s facial expressions (Cameron et al., 2019), or whether the person to empathize with is a human or an animal (Cameron et al., 2022). However, our paper will extend this paradigm to a novel context—the willingness to empathize when the target is either a single person or a group of people. This study will therefore contribute to the understanding of how people assess and react to opportunities to empathize with others, with a particular focus on what happens when the target goes from being an individual to being a group. This is especially relevant to investigate as previous research has shown that people perceive (Hamilton and Sherman, 1996) and respond (Butts et al., 2019; Västfjäll et al., 2014) differently to groups than they do to single individuals. For example, both affect and monetary donations are sometimes higher to a single individual than to a group (Kogut and Ritov, 2005a; Moche et al., 2022; Slovic, 2007). Thus, extending the EST paradigm to single vs. group recipients can have a bearing on how we understand people’s reaction and response to situations where not just single individuals, but several people are affected, such as in mass suffering situations (e.g., multiple victims of a disease, war, or natural disasters). Thus, differences in empathy choice between an individual and a group could possibly explain some of these previous findings but possibly also shed some light on how to resolve situations where our emotional response—and in turn, empathy—does not follow a linear increase for the number of people affected (i.e., psychophysical numbing; Slovic, 2007; Moche, 2022).

### Empathy

The term empathy today applies to several phenomena (Batson, 2009) and many definitions have been offered. In a recent review of reviews of how empathy has been defined, the authors concluded that there has been progress during the past decades in defining the concept, with many review papers highlighting four overlapping aspects of empathy (Eklund and Meranius, 2021). The four aspects of empathy were there defined as (1) *understanding*, (2) *feeling*, and (3) *sharing in another person’s world,* while (4) *maintaining a self-other differentiation.* In the empathy choice paradigm adapted in this paper, the focus in on the third aspect—the sharing in another person’s world. This has also been suggested to be the core of a standard definition of empathy (although this aspect also requires an understanding, or identification, of the other person’s world; Coll et al., 2017).

Further, the two central parts of empathy are the emotional sharing part (also referred to as affective empathy), meaning to subjectively experience and share in another’s psychological state or feelings (i.e., the second and third aspects in the definition above), and the perspective-taking part (also referred to as cognitive empathy), meaning to identify and understand another person’s feelings and perspective from an objective stance (i.e., the first and fourth aspects in the definition above; Decety and Cowell, 2014; Jeffrey, 2016). Although these two parts are interconnected in an empathic response (Coll et al., 2017), in our study, we focus primarily on empathy as experience sharing, since it is often at the center of debates about empathy (Bloom, 2017; Västfjäll et al., 2017), but importantly, because it is central in the methodological approach used by Cameron et al. (2019, 2022), which we apply. In the EST, participants who have chosen the empathy choice are instructed to *share in the feelings* of the target person (even though other facets of empathy can also become active in this task, as mentioned above, and also noted by the original authors; Cameron et al., 2019). Henceforth, we will use the word “empathy” as short for sharing internal experiences, measured as the number of empathy choices in the EST.

#### Effort and empathy

Although empathy is often considered important for altruism and social cognition (e.g., Batson, 2010), people also avoid empathizing when perceiving it as costly (Cameron et al., 2019, 2022). Previous research showed that empathy can be perceived as costly in different ways, such as when it includes an economic cost, requires time, or creates emotions of negativity (Andreoni et al., 2017; Cameron et al., 2016). The line of research using the EST has also identified that when people are given the choice to empathize with a single unknown individual, the majority decide to avoid it by staying objective, while experiencing high levels of cognitive cost and effort (Cameron et al., 2019, 2022).

According to the *motivated empathy perspective* (Cameron, 2018; Zaki, 2014), the reason why people decide to empathize or turn away in a particular situation depends on their assessment of the costs and benefits of empathizing. These cost–benefit assessments may involve financial, material, emotional, and social costs and rewards (e.g., reputation), but also cognitive factors, such as effort or ease felt by empathizing (Cameron, 2018; Zaki, 2014). The cost/benefit assessment can affect whether a person decides to approach (vs. avoid) empathy by using emotion regulation strategies (Gross and Thompson, 2007). In this study, we will investigate the cognitive and emotional factors of ease, effort, and distress associated with the act of empathizing. Further, we also look at participants’ perceived efficacy when performing the tasks, since this can be relevant for the experienced effort.

The emotional regulation strategy in focus for the EST is situational control. According to emotion regulation theories, people use strategies to adjust their emotions, such as how to express or experience them (Gross and Thompson, 2007). Situational control, as the specific strategy tested in the EST, involves adjusting one’s behavior or actions to make it more (or less) likely to find oneself in a situation that will give rise to desirable (or undesirable) emotions. This can for example entail keeping a distance from volunteers in the streets looking for donations or switching the channel to avoid hearing news about groups of people suffering. In our study, we investigate how participants exercise this strategy when empathizing with others, when “others” implies either a single individual or a group of individuals.

### Empathizing with individuals and groups

People seem to perceive individuals and groups differently, which can affect the choice to empathize with either one of them. For example, Hamilton and Sherman (1996) found that a single individual is viewed as more concrete, unitary, coherent, consistent, and entitative than a group. Thus, when choosing to empathize with a single individual versus a group, the results could go in two opposite directions.

1. One of the possible directions is that people will empathize more with a group than with an individual. As mentioned, an individual is perceived as a more coherent and consistent unit, leading to individuals eliciting more attention, elaborate processing, perspective-taking, and affect compared to a group of people (Hamilton and Sherman, 1996). Thus, the extra demand on information processing for an individual might elicit less willingness to empathize compared to a group. Further, Kogut and Ritov (2005b) found that participants seeing a single identified victim expressed significantly more distress than participants seeing a group of identified victims. Feelings of distress may evoke an egoistic motivation to reduce one’s aversive arousal (Cialdini et al., 1987), possibly leading people to turn away from what causes these emotions and thereby lead to less willingness to empathize with an individual compared to a group. Similarly, according to findings on compassion fade, people presented with one victim respond with more affect than those being presented with a larger number of victims (Butts et al., 2019; Moche et al., 2022; Slovic, 2007; Västfjäll et al., 2014). Last, it might be harder to empathize with an individual because of less external information. A group can give more context information and thereby make empathizing less effortful and error-prone (Dunn et al., 2017). In sum, these findings suggest that it is more cognitively and emotionally demanding to empathize with an individual, which would mean that people are more likely to empathize with a group than with an individual.
2. The other possible direction is that people will empathize more with an individual compared to a group. For example, as one identified individual is more likely to arouse affective reactions than a group of individuals (Kogut and Ritov, 2005b; Västfjäll et al., 2014), it could be argued that empathizing with the individual would be experienced as easier since it happens more naturally. Further, a group of people has the potential to elicit stronger emotional responses than a single individual due to the overwhelming amount of affective information. Because of this, Cameron and Payne (2011) suggested that our emotion regulation system proactively downregulates our emotional response when introduced to an increased number of victims (but see also Hagman et al., 2022). Thus, avoiding to empathize with the group can be viewed as an active attempt to regulate and lessen emotions that are seen as cognitively or emotionally costly. Last, there is also a greater risk of making empathic errors in judgment of a group’s inner emotional state compared to a single individual’s emotional state, which also supports this direction (Dunn et al., 2017). In sum, these lines of reasoning would mean that participants more often would choose to empathize with the individual than the group. To be able to arbitrate between these two possible predictions, we will directly pit empathy choice between individual and group targets against each other.

### Hypotheses

First, we expect that empathy is experienced as more cognitively costly than staying objective, and therefore the modal response in the EST will be to stay objective, in both the group and the single condition (hypothesis 1). This hypothesis is based on the findings from Cameron et al. (2019, 2022), who found that people are more likely to choose to stay objective than to empathize in the EST and that this is due to the increased cognitive costs associated with empathy choice. A possibly important distinction here is that avoiding empathy does not necessarily equate to staying objective in a real-life situation (e.g., one could focus on something completely else than the other person to avoid empathy), but since these are the two options pinned against each other in this particular paradigm and in the EST that we employ, we focus on these.

Second, as the EST-studies conducted so far not has compared empathy choices for single individuals with groups, and as outlined above, there are two possible directions of hypotheses regarding this comparison: A greater proportion of participants will choose to empathize in the group condition compared to the single condition (Hypothesis 2a), or a greater proportion of participants will choose to empathize in the single condition compared to the group condition (Hypothesis 2b). These hypotheses, along with our methodological approach and analyses were pre-registered through AsPredicted,^1^ before data collection.

## Methods

### Participants

Using G*Power, we calculated the number of participants needed in the study with our preferred power (0.80) and effect size (*f* = 0.2), yielding 266 participants. Taking possible dropouts into account, we aimed to recruit around 280–300 participants. Participants had to be American, over 18 years old, and speak English fluently. A total of 310 people from Prolific participated in the survey. Exclusion criteria were a failed attention check (*N* = 12) or not following the instructions of the task (*N* = 2), for example by using nonsense words (e.g., “cool whatever bro”). The final data set consisted of 296 participants (48% men, 50% women, 2% other, *M_age_* = 39.2, *SD* = 15.09). 55.1% of the participants held a bachelor’s degree or higher.

### Design

The study had a 2 (Card deck: feel vs. describe) × 2 (Picture block: single individual vs. group of individuals) within-subject design. Participants performed a modified version of the EST (Cameron et al., 2019, 2022), where they in addition to only making choices regarding one individual also made choices about a group. All participants also rated a number of items (described below). Completing the full survey took about 28 min and each participant was monetarily compensated for taking part in the experiment. The survey stimuli and exact instructions can be found in the Supplementary materials.

### Procedure

The study started with a short introduction and a consent form. Subsequently, participants answered an attention check, followed by the EST. The survey consisted of two main blocks of 20 different sets of pictures each. One block had pictures of a single individual and one block had pictures of a group of individuals. The pictures used in the two conditions were all carefully selected from a website selling stock photos and vectors.^2^ When choosing pictures for the different blocks we opted for pictures with both men and women, with different ethnicities, from a similar age group (i.e., 30–40-year-olds), and with neutral facial expressions. These choices were made to attempt to control for things like effects of experienced racial in-group on empathy (Xu et al., 2009) and how different facial expressions might affect the tendency to empathize (Eisenberg, 1986). The choice to go for neutral facial expressions was primarily based on Cameron et al. (2022), who used neutral facial expressions in their studies—with similar results in empathy choice as when using more emotional facial expressions (i.e., happy or sad facial expressions; Cameron et al., 2019). We also decided to keep all facial expressions neutral to eliminate variations, such as how different facial expressions might affect the tendency to empathize. Last, neutral facial expressions were also chosen to minimize stimulus differences in the pictures between the individual task and the group task. However, it was easier to find pictures of neutral faces for the single individual block than the group block, since pictures of groups of people tended to show a variation of facial expressions. This resulted in a broader range of emotion displayed in the pictures of the groups compared to the individuals.

**Figure 1:**
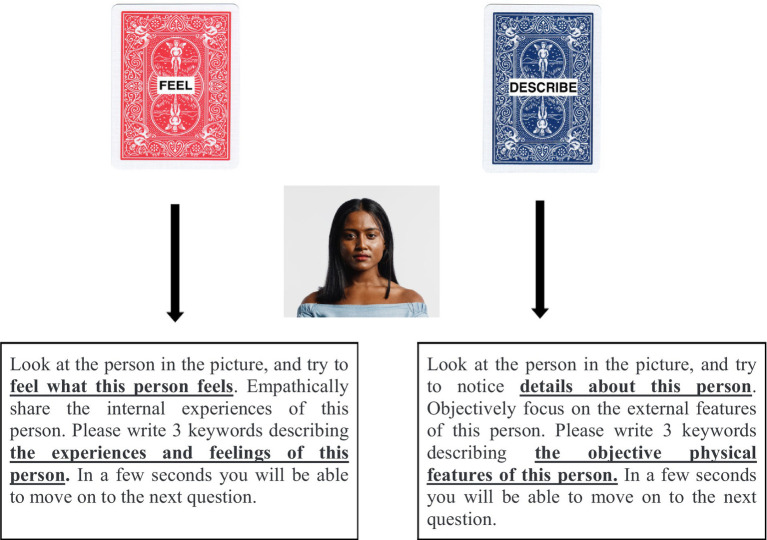
A schematic overview of survey flow for the individual block. Participants chose “feel” or “describe” and were then shown the picture with the instructions paired with their choice of card deck. Adapted with permission from https://www.123rf.com.

To control for order effect, the starting block (individual vs. group) was randomly chosen. Each block had specific instructions and a test trial for participants to practice on (see Supplementary materials). If participants answered wrongly during the test trial, they were given two additional chances to answer to make sure they understood the instructions. Then, participants were given the choice to pick one out of two decks of cards, one named “feel” (i.e., to empathize) and one named “describe” (i.e., to stay objective). The instructions were the same in both the individual block and group block, requesting them to “choose one of the decks.” i.e. feel or describe. After making this choice, participants saw a picture (either a single individual or a group of individuals, depending on which block they were in) and were asked to write down three keywords that either described the inner state (instructions for the feel deck) or the external features (instructions for the describe deck) of the individual(s). See Figure 1 for a visualization of the task. Participants were able to move on to the next deck of cards after 8 s. This time limit was added to limit the risk that participants were disengaged in the task and just went through the survey without taking the time to think. A time limit was also used in the study by Cameron et al. (2019, 2022). The participants were then repeatedly presented with the “feel” and “describe” decks of cards for a total number of 20 sets, showing different pictures in each set.

After completing a block, participants were asked to answer a set of questions from the NASA Task Load Index (Hart and Staveland, 1988) assessing cognitive effort, aversion, and efficacy, followed by an additional question to assess distress. Participants were asked to rate the questions with regards to the previously completed deck. This was done twice after each block, once regarding the feel deck, and once regarding the describe deck. When the questions were all rated, the first block was finished and participants could move on to the second block, which repeated the same procedure as mentioned above. Last, after the two main blocks were completed, participants answered a block of demographic questions. This included questions about age, gender, education, and place of residence.

### Measures

#### Primary measure

Our primary measure was the proportion of each chosen card deck, i.e., choosing to empathize or staying objective over the repeated trials. The choice proportion of the empathizing card deck, compared against chance (0.5), will be the main variable to test our first hypothesis (once for the individual block and once for the group block). For the second hypothesis, the proportion of choosing to empathize in the individual block will be compared to the proportion of choosing to empathize in the group block.

#### Secondary measures

Our secondary measures were “cognitive cost” and “distress” related to the choice of card deck in the EST. Questions measuring cognitive cost were taken and adapted from the NASA Task Load Index (Hart and Staveland, 1988). These measures were rated on a 5-point Likert scale ranging from 1 (=*Very low*) to 5 (=*Very high*). The two questions assessing effort concerned mental demand (“*How mentally demanding was this deck?”*) and effort (*“How hard did you have to work to accomplish your level of performance with this deck?”*), which we, using the approach from Cameron et al. (2019), combined. All samples on effort showed a good internal consistency with results above 𝛼 = 0.80. One question measured aversion (*“How insecure, discouraged, irritated, stressed, and annoyed were you by this deck?”*), and one question measured efficacy (*“How successful were you in accomplishing what you were asked to do in this deck?”*). As for the question on distress, it was taken and adapted from Kogut and Ritov (2005b). It was measured on a 7-point Likert scale ranging from 1 (*=Very low*) to 7 (*=Very high*). The question was: *“How worried, upset and sad were you when seeing each person/the groups of people?.”* For each of four secondary measures, the mean will be compared between the feel deck vs. describe deck, as well as between the individual vs. group block (and possible interactions).

## Results

### Primary analyses

In our primary analysis, where we wanted to investigate whether the choice to empathize would be avoided most often, we conducted two one-sample *t*-tests. These t-tests compared the choice of empathizing against chance (0.5) in each of the two blocks (group, individual; as was done by Cameron et al., 2022).^3^ See Table 1 for descriptives and the results of the *t*-tests. In the individual block, participants showed a significant preference to avoid empathy choice (i.e., choosing to stay objective more often). In contrast, participants in the group block showed a significant preference for the empathy choice compared to staying objective. Our first hypothesis, which stated that the modal response in the EST will be to stay objective (i.e., avoid empathizing), was therefore confirmed for the individual pictures, but not for the group pictures.

**Table 1:** Tests of empathy choice against chance by task block.

Picture	*M*	*SD*	*t*	*p*	95% CI difference	Hedges’ g
Group of individuals	0.53	0.28	1.2	0.047	[0.00, 0.06]	0.12
Single individual	0.34	0.27	−10.17	<0.001	[−0.19, −0.13]	−0.59

Df of t is 295. Choice (M) refers to the proportion of times the card deck “feel” was selected out of the total number of choices (20) presented to the participant. Hedges’ g reflects effect deviation of choice from chance (0.5) for one test.

Further, to explore if there was a significant difference between the two conditions (group, individual) in empathy choice, as highlighted in our second hypothesis, we conducted a paired sample t-test. The result showed a significant difference in empathy choice, *t*(295) = 12.04, *p* < 0.001, *g* = 0.28. Participants decided to empathize with the group to a greater proportion compared to the individual. This thus supports hypothesis 2a.

### Secondary analyses

Second, we wanted to explore possible explanations to these result by looking at the ratings of effort, aversion, efficacy, and distress. We conducted three separate two-way ANOVAs for effort, aversion, and efficacy, respectively, with the independent variables of card deck (feel, describe) and picture block (individual, group). The data regarding the fourth dependent variable, distress, was not normally distributed and therefore did not fit the criteria for conducting a parametric analysis. The Friedman test was therefore used as a non-parametric replacement for the ANOVA. The descriptive statistics of the variables can be seen in Table 2.

**Table 2:** Mean and standard deviation of cognitive cost variables (effort, aversion, efficacy) and the distress variable for describe and feel choices for both the single and group blocks.

	Single individual		Group of individuals	
	Describe	Feel	Describe	Feel
Effort	2.74 (1.17)	3.56 (1.11)	3.30 (1.11)	3.46 (1.07)
Aversion	2.04 (1.11)	2.60 (1.26)	2.56 (1.37)	2.54 (1.30)
Efficacy	3.87 (0.98)	3.37 (1.14)	3.57 (1.06)	3.62 (1.04)
Distress	2.00 (1.39)	2.51 (1.61)	2.21 (1.56)	2.59 (1.75)

#### Cognitive cost

##### Effort

The two-way ANOVA for effort showed two significant main effects and one significant interaction effect. The significant main effect of deck showed that participants rated the feel deck as significantly more effortful than the describe deck, *F*(1, 295) = 62.44, *p* < 0.001 *η_p_*^2^ = 0.18. There was also a main effect of picture, where participants rated the group block as significantly more effortful than the individual block, *F*(1, 295) = 21.74, *p* < 0.001. *η_p_*^2^ = 0.07. Last and more importantly, there was a significant interaction effect, *F*(1, 295) = 49.83, *p* < 0.001, *η_p_*^2^ = 0.15. A Bonferroni-corrected pairwise comparison showed that participants rated the describe deck in the group block as more effortful compared to the individual block (*p* < 0.001), whereas there was no significant difference between blocks for the feel deck (*p* = 0.111). The pairwise comparison also showed that the feel deck was rated more effortful compared to the describe block, both in the individual (*p* < 0.001) and the group block (*p* = 0.031).

##### Efficacy

The two-way ANOVA for perceived efficacy showed a significant main effect and an interaction effect. The significant main effect of deck showed that participants rated significantly higher efficacy for the describe deck than the feel deck, *F*(1, 295) = 15.78, *p* < 0.001. *η_p_*^2^ = 0.05. No significant main effect was found regarding picture, *F*(1, 295) = 0.23, *p* = 0.632. More importantly, there was a significant interaction effect, *F*(1, 295) = 35.22, *p* < 0.001, *η_p_*^2^ = 0.11. A Bonferroni-corrected pairwise comparison showed that participants’ efficacy for the describe deck was significantly higher in the individual block than the group block (*p <* 0.001), whereas it for the feel deck was significantly higher in the group block than the single block (*p* < 0.001).

##### Aversion

The two-way ANOVA for perceived aversion showed two significant main effects and an interaction effect. The significant main effect of deck showed that participants rated the feel deck as significantly more averse than the describe deck, *F*(1, 295) = 22.11, *p* < 0.001, *η_p_*^2^ = 0.07. The significant main effect of picture showed that participants rated the group block as significantly more averse than the individual block, *F*(1, 295) = 18.68, *p* < 0.001, *η_p_*^2^ = 0.06. More importantly, we found a significant interaction effect, *F*(1, 295) = 38.44, *p* < 0.001, *η_p_*^2^ = 0.115. A Bonferroni-corrected pairwise comparison showed that participants rated the describe deck as significantly more averse for the group block than the individual block (*p* < 0.001), whereas there was no significant difference between blocks for the feel deck (*p* = 0.361).

#### Distress

Since the data for the distress variable was not normally distributed, we conducted a Friedman test, which is a non-parametric equivalent to a repeated-measures ANOVA. The results showed a significant difference between groups, *χ*^2^(3) = 59.38, *p* < 0.001. A Bonferroni corrected pairwise comparison showed that distress was significantly higher for the feel deck compared to the describe deck, in both the individual block (*p* < 0.001), and the group block (*p* < 0.001). This indicates that the feel deck in general elicited higher levels of distress than the describe deck. There was no significant difference between the group and individual block in rated distress for the describe (*p* = 0.166) or feel deck (*p* = 0.861).^4^

### Exploratory analyses

To try to understand the relationship between our secondary variables, we conducted a Pearson’s correlation. The correlations are found in Table 3. All were significantly correlated, ranging from a small to large effect size (Cohen, 1988).

**Table 3:** Pearson’s correlations between cognitive cost variables and distress.

Variable	M	SD	1	2	3
1. Effort	3.27	1.16			
2. Aversion	2.43	1.28	0.591**		
3. Efficacy	3.61	1.07	−0.198**	−0.295**	
4. Distress	2.33	1.60	0.344**	0.443**	−0.175**

**p < 0.01.

## Discussion

This paper has used an adapted version of EST (developed by Cameron et al., 2019) to investigate how people choose to empathize when presented with a single individual and a group of individuals. We also investigated how experienced cognitive costs and distress were associated with empathy choice. First, we hypothesized that participants more often would avoid empathizing, in both the group and the individual block. This was confirmed in the individual block, but not in the group block. That is, participants chose to stay objective significantly more often than empathizing for individuals, whereas it was the opposite for the group. Further, we found that there was a preference to empathize with the group over the individual, in line with hypothesis 2a. Last, our results for cognitive cost and distress were partially in line with previous findings (Cameron et al., 2019, 2022), such that empathizing was more effortful, aversive, and distressing than staying objective.

First, when the decision to empathize concerned a single individual, significantly more people decided to stay objective. Thus, our findings replicate those of Cameron et al. (2019). However, this was not the case for a group of individuals. Rather, the opposite was true then, showing that significantly more people decided to empathize with the group over staying objective. More specifically, participants chose the empathy choice on average in 34% of the trials in the single condition, whereas the same participants on average chose the empathy choice in 53% of the trials in the group condition. Although not a strong preference for empathizing (53%), it was still significant and in the opposite direction of our hypothesis and previous research. Further, when specifically comparing the empathy choice for the individual and the group block, it also showed that participants chose to empathize with the group significantly more often than they did for an individual, which taken together supports hypothesis 2a. Possible reasons for these results are discussed related to our secondary measures below.

In line with Cameron et al. (2019, 2022), we further found that participants experienced the feel deck as significantly more effortful and aversive than the describe deck. Interestingly, participants in the group block rated the feel deck as more effortful than the describe deck but they still chose it more often. This result contrasts with the findings of Cameron et al. (2019) who hypothesized that due to its cognitive costs, higher experienced effort would result in participants choosing to avoid empathizing more often. A possible explanation for our result is the increased amount of information in the group condition. That is, the increased external information in the group condition can lead to more errors in empathic judgment and hence make it feel more effortful (Dunn et al., 2017). At the same time, the group also provides more context information and clues (e.g., body language) to use when processing people’s inner emotional state (Clark et al., 2020), possibly explaining why participants in the group condition still chose to empathize more often. Last, the increased number of external stimuli could make it harder for the participant to process the extra information, hence, rating the describe deck as more effortful for the group than the individual deck. Thus, these results can be understood in relation to description preference. That is, what makes a target easy or difficult to describe? It should be relatively easy to describe the features of a single individual, especially when conveying a neutral facial expression. Supporting this, the mean rating for the describe deck of the individual was relatively small, suggesting participants did indeed experience it as rather effortless. However, it should be harder to describe a group, or a single individual conveying a more emotional facial expression. Thus, the results regarding empathy choice and effort can perhaps best be understood in relation to description preference.

Further, participants rated themselves to be more efficacious in describing than empathizing. This is possibly due to less likelihood of error when describing objective features in a picture (Dunn et al., 2017). It can also be connected to the scores in effort, where higher efficacy could mean it was felt as less effortful. This is confirmed by the correlations showing a negative correlation between the two variables. Thus, when people feel themselves to be more efficacious, they rate the task as less effortful and aversive. Also, participants perceived themselves to have more efficacy in the individual block than in the group block when choosing to describe, whereas it was the opposite for empathizing (i.e., higher efficacy in the group than the individual block for empathizing). Again, this might be due to more external information in the group condition, making it harder to describe but easier to empathize. The results of efficacy might also explain why participants chose to feel more often in the group condition. That is, even if participants in the group condition felt more effort to empathize, people might have chosen to do what they felt they were good at, despite the effort it also took.

Looking at the distress variable, participants rated significantly higher levels of distress for the feel deck compared to the describe deck in both the group and individual block. This can be understood by previous research by Cialdini et al. (1987) where feelings of distress may evoke an egoistic motivation to reduce one’s aversive arousal, leading people to turn away from the stimuli causing distress. Regarding the group block, participants chose the feel deck more often even though they also rated it as eliciting higher levels of distress. These results are similar to the ones for effort and aversion (i.e., participants chose the feel deck more often even though they rated it as more effortful and aversive), evidenced by the positive correlations between the variables.

When looking closer at the feel deck, we found no significant difference in rated distress between the group and individual block with the non-parametric test. To some extent, this contrasts with the results of Kogut and Ritov (2005b) who found that an identified single victim elicited higher distress than a group—which we did not find. However, there are differences between our and their study, such as the context and level of identifiability. Further, our results showing no difference in distress between the group and individual block can possibly be understood by psychophysical numbing. That is, our emotional response to a group does not follow a linear increase from those to a single individual (Slovic, 2007). Thus, a group of people does not necessarily elicit higher levels of distress compared to a single person. However, we acknowledge that our research and that on psychophysical numbing also differ in context (i.e., whether there is a need for help). Also, the distress variable showed a floor effect. This might be due to the neutral faces without context in the blocks, which are less likely to evoke feelings of distress in comparison to, e.g., an identified victim described to be in need of help (Kogut and Ritov, 2005b; Moche et al., 2020, 2024; Västfjäll et al., 2014).

## Limitations and future studies

When choosing pictures to include, we opted for those with neutral faces. Further, the group pictures showed full bodies compared to the individual pictures with just a headshot. Just as Clark et al. (2020) mention, our emotional interpretations do not exist in a vacuum of only facial expressions but instead happen in a combined evaluation of multiple contextual factors, such as body language and surroundings. This differing aspect may have influenced the results, but it is hard to say how. Another aspect worth mentioning is how neutral facial expressions may affect the tendency to empathize. Clark et al. (2020) argue that when there is an absence of contextual information other than the facial expression of a person, there is greater variance in the interpretation of an emotion. The neutral faces can thus make it hard to identify (or understand) the person’s emotional state and subsequently make it harder to share in those feelings (Coll et al., 2017). A neutral face can be argued to give less information than a smiling or sad face, even though neutral faces still can convey affective information (Gasper, 2018). Often in empathy research, negative facial expressions are used. However, in Cameron et al. (2019), the pictures used were portraits with sad or happy faces and in Cameron et al. (2022) they portrayed neutral facial expressions—but both still gave the same result (i.e., participants avoided empathizing more). However, the faces of children in this context have not yet been studied (compared to adults, e.g., Moche and Västfjäll, 2021), which may be of interest for future studies.

In one of the studies by Cameron et al. (2022), participants were instructed to choose to empathize with an individual or an animal, but instead of putting them in different blocks to be rated separately, they were pinned against each other (e.g., choose to empathize with the individual or the animal). This differs from the present study. Here, participants were never directly asked if they preferred to empathize with the individual or the group. However, since this was a within-subject design, participants saw both options in a counter-balanced order. Nevertheless, this version could be tested in a future study to see how the results compare against each other, and if ratings of cognitive cost or distress would change.

Last, adding more context to the EST can be used to see the boundary condition on empathy avoidance. To further the cost–benefit analysis that is discussed, the added contextual information can also vary, such as information about the economic, material, and social costs to examine the robustness of the cognitive cost variable.

## Conclusion

The purpose of this study was to replicate and extend the findings of Cameron et al. (2019, 2022) by testing if the choice to empathize differs between a group and an individual, and if so, how and why. Our results in the individual condition were similar to previous findings, such that people more often chose to stay objective over empathizing. Empathizing was also rated as more cognitively costly and distressing. However, participants in the group condition more often chose to empathize over staying objective, despite also rating it as more effortful and distressing. This might be due to higher ratings of efficacy at empathizing in the group block compared to the individual block, but also due to more contextual information in the group condition. This study shows that it may not only be the cognitive cost variable that affects the decision to empathize, but to a degree, so may feelings of distress. Our study is the first to compare empathy choice for individuals and groups and the results provide another nuance in explaining empathy and what may or may not motivate it.

## Data Availability

The datasets presented in this study can be found in online repositories. The names of the repository/repositories and accession number(s) can be found at: OSF-website (https://osf.io/7vskg/).

## Footnotes

^1^ https://aspredicted.org/L6D_YQL

^2^ https://www.123rf.com/

^3^The pre-registration made for this study stated that we would conduct three separate two-way ANOVAs and one sample t-test. However, we realized that we in fact needed to do t-tests in each block (group, individual) to compare them with chance, and then a paired t-test to test if the difference in empathy choice between the blocks (group, individual) was significant.

^4^For comparison, we also did four Wilcoxon signed rank tests. The Wilcoxon signed rank tests showed the same comparisons as the Friedman test to be significant. It also found a significantly higher rating in distress for the describe deck in the group block compared to the individual block (*p* = 0.007). Since the Wilcoxon test is slightly less conservative than the Friedman test, this result is not surprising. Significance levels for the Wilcoxon tests were Bonferroni-corrected to reduce the risk of type I error.
