# Supplementary material for: To empathize with a group or an individual? Investigating the role of cognitive cost and distress in empathy choice
Source: Front Psychol. 2025 May 7;16:1519113. doi: 10.3389/fpsyg.2025.1519113 (PMC12092378; doi:10.3389/fpsyg.2025.1519113)
Supplement: Supplementary file 1 [file Supplementary_file_1.docx]

Supplementary materials –

To empathize with a group or an individual? Investigating the role of cognitive cost and distress in empathy choice

Content

[*Stimuli and descriptions used in the experiment* 1](#_Toc139294891)

[1. *Initial description of the task* 1](#_Toc139294892)

[*2.* *Test trials* 3](#_Toc139294893)

[*3.* *Instructions given after each empathy choice made in the blocks* 4](#_Toc139294894)

# *Stimuli and descriptions used in the experiment*

Below are the main descriptions and instructions given to participants in the experiment. The instructions for the measures are also shown below.

1. *Initial description of the task*

The description below was given at the start of both the individual block and the group block:

“In this task, you will complete a series of trials. On each trial, you will see two decks of cards: the blue deck will always be labeled “DESCRIBE” and the red deck will always be labeled “FEEL”. You should choose between these decks. Once you choose a deck, you will then see an image of a single person [Group block: a group of people]. Depending on which deck you have chosen, you will be given one of two possible sets of instructions.

If you choose from the deck labeled “DESCRIBE”, you will be told to be objective and focus on the external features and appearances of the person in the image. When completing this kind of trial, try to be as objective as possible. To be objective, do not let yourself get caught up in imagining what this person feels.

If you choose from the deck labeled “FEEL”, you will be told to have empathy and focus on the internal feelings and experiences of the person in the image. When completing this kind of trial, try to feel as much empathy as possible. To be empathic, let yourself get caught up in imagining what this person feels.

You are free to choose from either deck on any trial, and should feel free to move from one deck to the other whenever you choose. If one deck begins to seem preferable, feel free to choose that deck more often. Overall, this task will take the same amount of time regardless of which deck you choose.”

1. *Test trials*

After the instructions, participants underwent two test trials, given at the start of each block. Below are the trials shown to participants, first the two test trials presented in the individual block, followed by the two test trials in the group block:

Which of the following is an appropriate response on trials with one person where you are told to be empathic?

- Smiling, white, woman
- Happy, interested, curious
- Woman, happy, blond

Which of the following is an appropriate response on trials with images of one person where you are told to be objective?

- Interested, woman, white
- Woman, young, blond
- Happy, curious, blond

Group block

Which of the following is an appropriate response on trials with a group of people where you are told to be objective?

- Five people, mixed gender, white
- Irritated, young, brunettes
- Happy, energetic, blondes

Which of the following is an appropriate response on trials with a group of people where you are told to be empathic?

- Smiling, white, women
- Women, happy, blond
- Happy, interested, curious

1. *Instructions given after each empathy choice made in the blocks*

After the test trials, participants were presented with a choice to choose a deck before seeing one picture at the time (either an individual or a group of individuals). Below are the instructions given following a feel-deck choice and after, the instruction given following the describe-deck choice:

Look at the person in the picture, and try to feel what this person feels. Empathically share the internal experiences of this person. Please write 3 keywords describing the experiences and feelings of this person. In a few seconds you will be able to move on to the next question.

________________________________________________________________

Look at the person in the picture, and try to notice details about this person. Objectively focus on the external features of this person. Please write 3 keywords describing the objective physical features of this person. In a few seconds you will be able to move on to the next question.
 ________________________________________________________________
